# A LATENT CLASS APPROACH TO UNDERSTANDING HOMELESSNESS AMONG OLDER ADULTS

**DOI:** 10.1093/geroni/igae098.0396

**Published:** 2024-12-31

**Authors:** Anita Souza, Jenny Tsai, Kenneth Pike

**Affiliations:** University of Washington, Seattle, Washington, United States; University of Washington, Seattle, Washington, United States; University of Washington, Seattle, Washington, United States

## Abstract

The complex characteristics that differentiate adults over 50 experiencing homelessness are understudied. Latent class analysis (LCA) is a novel approach to identifying unobservable subgroups within populations. Housing providers have little information to adopt tailored service approaches. Capitalizing on the strength of LCA, this paper identifies the heterogeneity within older adults utilizing housing. Using a retrospective cohort study design, we extracted data from the Homeless Management Information System to describe the health conditions of adults utilizing services of a leading housing provider in Washington. These data captured developmental disability, physical health, disabling conditions, chronic conditions, mental health problems, and substance use. Among 1,971 homeless individuals, 76% were male, 49% white, and 70% homeless 6 months or more. Forty percent were 50 years or older; 17% were veterans. Probability calculated by LCA found that a 4-class solution best represented the data (fit indices: AIC=11,688, BIC=11839, entropy=0.85). Class 1 (n=519) reported higher than average proportions of mental health problems and substance abuse along with average proportions of members with a chronic health condition and slightly higher than average proportion of members with a disabling condition. Class 2 (n=254) was dominated by individuals with a disabling condition and physical disability. Class 3 (n=827) reported the lowest proportion of members with all conditions. Class 4 (n=371) was higher than average on all conditions. This knowledge builds a foundation for further research and policy endeavors to support older adults experiencing homelessness.

